# Driving Advice on Discharge: An Audit of Compliance With DVLA and GMC Standards in Psychiatric Care

**DOI:** 10.1192/bjo.2026.11652

**Published:** 2026-06-30

**Authors:** Faisal Alam, Abdul Rhouma, Sadia Nawaz, Assad Awan, Min Sein

**Affiliations:** LSCFT NHS Foundation Trust, Preston, United Kingdom

## Abstract

**Aims:**

Psychiatric conditions and psychotropic medications can significantly impair driving ability through sedation, impaired judgment, or delayed reaction times. According to DVLA and GMC standards, clinicians must advise patients when their mental state or medication may affect driving, inform them of their duty to notify the DVLA, and clearly document this advice. This audit assesses compliance with these requirements on two psychiatric inpatient wards.

**Methods:**

A retrospective review of 20 discharge summaries (10 from Edisford Ward, 10 from Hyndburn Ward at the Royal Blackburn Hospital) dated up to 15/09/2025 was conducted. Data were extracted under the following domains: diagnosis, driving status, medication class, evidence of driving advice on discharge, and evidence of DVLA contact on behalf of the patient.

**Results:**

Across both wards, 20 patients were reviewed in total. The vast majority (n=19, 95%) were prescribed psychotropic medications that may impair driving, including antipsychotics, antidepressants, benzodiazepines, and mood stabilisers.

Only 1 patient had documented evidence that driving advice was given at discharge. No cases recorded that the clinician contacted the DVLA on behalf of the patient, even where severe psychiatric illness (e.g. schizophrenia, bipolar disorder, or mania) was present.

**Conclusion:**

**Discussion:**

The findings demonstrate a clear gap in documentation regarding driving advice in psychiatric inpatient discharges. Despite all patients being prescribed medication with potential to impair driving, almost none had documented advice, and there was no evidence of clinician escalation to the DVLA.

**Recommendations:**

Provide ward-based education sessions on DVLA standards and GMC responsibilities. Ensure driving advice is provided to patients on discharge and is clearly documented. Re-audit after 1 month to assess improvement.

**Conclusion:**

This audit highlights poor compliance with national guidance regarding driving advice and documentation at discharge. Improved awareness and systematic prompts are required to ensure patient and public safety and to maintain medico-legal standards.

